# TiO_2_ Immobilized on Manihot Carbon: Optimal Preparation and Evaluation of Its Activity in the Decomposition of Indigo Carmine

**DOI:** 10.3390/ijms16011590

**Published:** 2015-01-12

**Authors:** Cynthia M. Antonio-Cisneros, Martín M. Dávila-Jiménez, María P. Elizalde-González, Esmeralda García-Díaz

**Affiliations:** 1 Centro de Química -ICUAP, Universidad Autónoma de Puebla; Ciudad Universitaria, Edif. H103, Puebla 72570, Mexico; E-Mails: cantonio@unpa.edu.mx (C.M.A.-C.); esmeralda.garcia.uap@gmail.com (E.G.-D.); 2 Instituto de Agroingeniería, Universidad del Papaloapan, Campus Loma Bonita, Oaxaca 68400, Mexico; 3 Facultad de Ciencias Químicas, Universidad Autónoma de Puebla, Ciudad Universitaria, Edif. H105, Puebla 72570, Mexico; E-Mail: mdavila.uap.mx@gmail.com

**Keywords:** carbonization, Manihot, anatase, photocatalyst, indigo carmine, Taguchi, photoproducts, identification

## Abstract

Applications of carbon-TiO_2_ materials have attracted attention in nanotechnology due to their synergic effects. We report the immobilization of TiO_2_ on carbon prepared from residues of the plant Manihot, commercial TiO_2_ and glycerol. The objective was to obtain a moderate loading of the anatase phase by preserving the carbonaceous external surface and micropores of the composite. Two preparation methods were compared, including mixing dry precursors and immobilization using a glycerol slurry. The evaluation of the micropore blocking was performed using nitrogen adsorption isotherms. The results indicated that it was possible to use Manihot residues and glycerol to prepare an anatase-containing material with a basic surface and a significant S_BET_ value. The activities of the prepared materials were tested in a decomposition assay of indigo carmine. The TiO_2_/carbon eliminated nearly 100% of the dye under UV irradiation using the optimal conditions found by a Taguchi L4 orthogonal array considering the specific surface, temperature and initial concentration. The reaction was monitored by UV-Vis spectrophotometry and LC-ESI-(Qq)-TOF-MS, enabling the identification of some intermediates. No isatin-5-sulfonic acid was detected after a 60 min photocatalytic reaction, and three sulfonated aromatic amines, including 4-amino-3-hydroxybenzenesulfonic acid, 2-(2-amino-5-sulfophenyl)-2-oxoacetic acid and 2-amino-5-sulfobenzoic acid, were present in the reaction mixture.

## 1. Introduction

Semiconductor photocatalytic processes are emerging as a water and wastewater treatment technology under the “zero waste” scheme [1]. Concerning semiconductors, TiO_2_ is the most important material in environmental purification systems due to its photoelectrochemical and photo-induced superhydrophilic properties, as reviewed by Ochiai and Fujishima [2]. Combinations of TiO_2_ and carbon represent interesting materials and are under active study [3]. The most extensively studied combinations include TiO_2_ nanoparticles coated with carbon, carbon impregnated with TiO_2_ and mechanical mixtures of TiO_2_ and carbon. Carbon-TiO_2_ materials are interesting because (a) the materials are more easily recovered after use in a wide range of applications [4] and (b) their texture [5] and pH [6] are different from those of the starting materials.

Applications of photocatalytic carbon-TiO_2_ have attracted widespread attention due to the decomposition activity of the material toward effluents [7]. Whereas carbon doping promotes band-gap narrowing, the presence of carbon in TiO_2_ samples promotes adsorption. A critical review by Lim clearly illustrated several of the practical issues surrounding the preparation of carbon-TiO_2_ composites [8]. These materials can be designed with carbon powders, fibers, nanotubes, fullerenes or graphene [3]. Noteworthy studies of Zhang *et al.* did not show any difference between the photoactivity displayed by composites containing different allotropic forms of carbon, both in gas and liquid phase degradations [9,10]. However, the different carbon-TiO_2_ systems enhance in a different way their photocatalytic activity through one or all of the three primary mechanisms: minimization of the electron-hole recombination rate, band gap tuning and improvement of adsorption sites [3]. Because we used a biomass carbonized at relatively low temperature, we have purposely confined the discussion to activated carbon. In most of the investigations where carbon-TiO_2_ materials have been prepared, the authors have preferred to use commercial activated carbon [7,11,12,13]. Research on the thermochemical conversion of biomass to develop the carbonaceous component of a carbon-TiO_2_ composite is limited.

Biomass, such as bamboo leaves [14], pine sawdust [15], maize corncob residues [16], canola hull [17] and corn straw powder [18], has been used as a carbon precursor to prepare powdered carbon-TiO_2_ materials via the mixing and hydrothermal procedure. The carbon-TiO_2_ ratios were 1:0.01 [17,18], 1:0.1 [14,15,17] and 1:2 [18], and the carbonization temperature in these studies varied over a broad range from 400 °C [14] to 800 °C [16,18]. Cordero *et al.* used *Tabebuia pentaphylla* wood to produce carbon for use in the presence of TiO_2_ particles in a photocatalytic experiment [19]. In this investigation, no carbon-TiO_2_ composite was actually prepared. However, aggregation of nanoparticles of TiO_2_ on the prepared carbon samples was observed. Interestingly, the photocatalytic activity upon photodegradation of 4-chlorophenol was correlated with the texture and surface chemistry of the activated carbon that was added. The immobilization of TiO_2_ particles onto solid supports has been a key technical strategy for a cost effective solid-liquid separation. In this sense, this work aims to use as support a carbon material obtained from a no cost agricultural waste. Furthermore, the use of a carbonaceous support is justified because it has been proven that the delocalized conjugated π-structures of carbon materials promote a rapid photoinduced charge separation and slow the charge recombination rate in electron-transfer processes [2].

The methods used to load TiO_2_ onto carbon include four possible procedures as follows: (i) mixing activated carbon with a previously prepared TiO_2_ powder; (ii) coating carbon with films of TiO_2_ precursors using sol-gel and hydrothermal techniques or chemical vapor deposition; (iii) mixing a carbon precursor with a TiO_2_ sol-gel and *in situ* one-step carbonization/calcination and (iv) combining a carbon precursor with finished TiO_2_ particles followed by carbonization. Depending on the nature of the precursor, methods (iii) and (iv) may involve two hydrophilic raw components or one hydrophobic component and one hydrophilic component. Processes (i) and (ii) require the creation of real contact between the hydrophobic and hydrophilic components of the composite. The use of an additive acting as an intermicellar liquid may stabilize one hydrophilic (TiO_2_) component and one hydrophobic (carbon) component. However, little is reported on the use of additives in the preparation of carbon-TiO_2_ composites. One of our goals was to develop a rapid method for the optimum immobilization of moderate amounts of the anatase-phase of TiO_2_ on carbon using thermal processing (at 800 °C) of *Manihot* residues with the aid of glycerol. This material is referred to as TiO_2_/carbon. In addition, we assessed the performance of the prepared materials by employing them for the photocatalytic decomposition of indigo carmine (IC).

## 2. Results and Discussion

### 2.1. Texture of the Composites Obtained from Dry Mixtures

**Figure 1:**
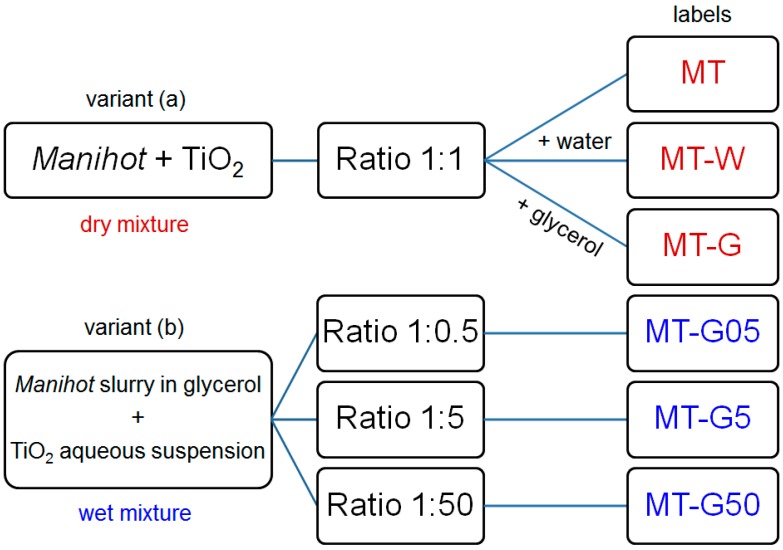
General scheme depicting two preparation variants and labels of the materials prepared from the *Manihot* residues at 800 °C.

The raw *Manihot* residues had an irregular elongated shape, which was retained in the carbonized samples. A preliminary trial consisted of mechanical dry mixing of the *Manihot* carbon C800 with TiO_2_ in a weight ratio 1:1. This corresponded to procedure (*i*) mentioned in the introduction. The resulting material exhibited a specific surface S_BET_ of 345 m^2^·g^−1^, ~60% microporosity and an average pore diameter of 7.4 nm. However, the TiO_2_ particles were not permanently immobilized on the carbon surface. This observation led us to develop two different one-step preparation methods using raw residues according to procedure (*iv*). The two variants of the preparation method are shown in Figure 1.

**Figure 2:**
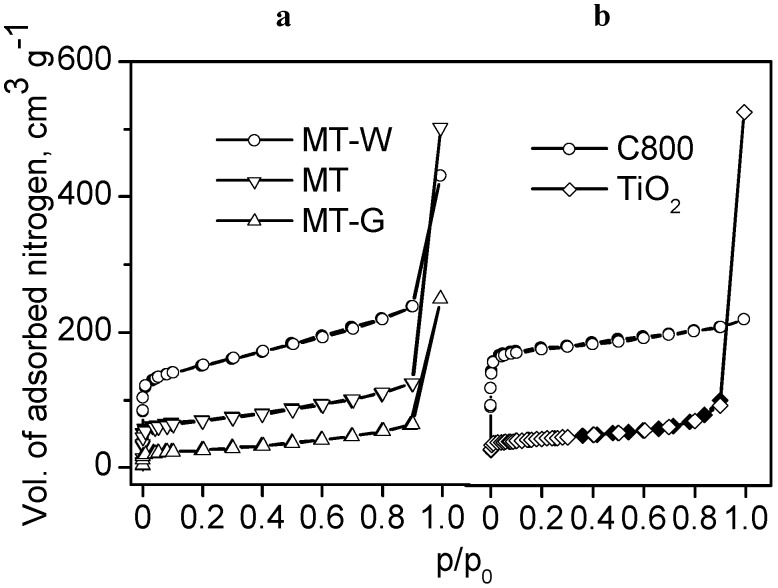
N_2_ adsorption (empty symbols) and desorption (filled symbols) isotherms at 77 K for (**a**) the materials prepared from dry mixtures of raw *Manihot* residues and TiO_2_ (variant (a) in Figure 1) and (**b**) *Manihot* carbon C800 (*d_p_* 0.25 mm) and the photocatalyst TiO_2_.

Figure 2a shows the nitrogen adsorption isotherms of the sieved samples of the MT, MT-W and MT-G composites prepared by dry mixing by variant (a), as shown in n Figure 1. As a reference, the N_2_ adsorption isotherms of the Manihot carbon C800 and commercial TiO_2_ are shown in Figure 2b. Table 1 lists the texture parameters of the precursors (TiO_2_ and *Manihot*) and the carbon obtained at 800 °C using the vascular system of the *Manihot* stems.

**Table 1:** Texture parameters of the precursors, carbon C800 and TiO_2_/C materials prepared in a 1:1 *Manihot*: TiO_2_ ratio by variant (a) as depicted in Figure 1, and the corresponding isotherms are shown in Figure 2.

Material		Specific Surface (m^2^·g^−1^)			V_μ_, cm^3^·g^−1^	V_tot_, cm^3^·g^−1^	Average Pore Diameter, nm
		BET	S_0_^t^ (%)	S_e_^t^			
Precursors	TiO_2_	58	-	-	-	0.678	-
	*raw Manihot*	72 ^a^	-	-	-	-	-
Carbon	C800	449	318 (71)	130	0.160	0.263	2.6
TiO_2_/C	MT	226	100 (44)	126	0.051	0.712	6.9
	MT-W	493	245 (50)	248	0.125	0.611	5.4
	MT-G	87	24 (28)	63	0.013	0.353	17.6

^a^ measured by the adsorption of methylene blue from solution.

A comparison of the texture parameters in Table 1 demonstrates the effect of the addition of water or glycerol on the dry Manihot + TiO_2_ mixture. The specific surface area obtained for composite MT (S_BET_ = 226 m^2^·g^−1^) increased when the sample was prepared in the presence of water (*i.e.*, MT-W, S_BET_ = 493 m^2^·g^−1^) and decreased in the presence of glycerol (S_BET_ = 87 m^2^·g^−1^). A comparison of the micropore surface S_0_^t^ (318 m^2^·g^−1^) of carbon C800 with that of MT-G (24 m^2^·g^−1^) indicated that glycerol partially inhibited micropore development because the contribution of the micropore surface S_0_^t^ to the total specific surface decreased from 71% in C800 to 28% in MT-G.

Without glycerol, the TiO_2_ nanoparticles settled on the raw *Manihot* residues prior to carbonization in MT and MT-W. The contribution of S_0_^t^ to S_BET_ was similar (*i.e.*, 44% and 50% in MT and MT-W, respectively). Because the values of microporosity were lower than those in carbon C800 (71%), the TiO_2_ nanoparticles also hindered the microporosity to some extent.

The prepared composites substantially exceeded the S_BET_ of bare TiO_2_ (58 m^2^·g^−1^). However, the total pore volume V_tot_, measured at p/p_0_ = 0.99 for the MT and MT-W samples was similar to that of the precursor TiO_2_ (0.678 cm^3^·g^−1^). The total pore volume of sample MT-G (0.353 cm^3^·g^−1^), which was prepared with glycerol, was comparable to that of carbon C800 (0.263 cm^3^·g^−1^). However, the glycerol prevented micropore formation, and the external surface provided the greatest contribution (72%) to the magnitude of the specific surface in MT-G. Nevertheless, the composites prepared by this methodology can be regarded as mesoporous materials with a broad pore size distribution because the average pore width varies within 5.4 ≤ D_p_ ≤ 17.6 nm (see Table 1).

The raw *Manihot* residues exhibited hydrophilic and nearly neutral characteristics with a pH_pzc_ of 6.7 [20], which is very close to the pH_pzc_ of anatase (6.5) [21]. Therefore, a certain chemical affinity can be expected between both materials.

However, the composites prepared using the dry mixture approach (variant (a) in Figure 1) did not exhibit a satisfactorily homogeneous aspect when they were observed under the microscope, which may be due to a “like agglomerates like” effect (*i.e.*, the attraction of TiO_2_ to TiO_2_ and carbon to carbon) during mixing. Furthermore, the addition of water in MT-W or glycerol in MT-G affected the texture properties of the composites very differently. Because the strongest effect occurred in the micropore volume of MT-G, the glycerol additive aids in the formation of a homogeneous mixture of raw *Manihot* residues and TiO_2_ and favors the development of the external surface (up to 72% in MT-G). Carbonization of glycerol itself yields a smooth carbonaceous surface and hinders the TiO_2_ nanoparticles from being trapped in the micropores, which improves exposure to irradiation.

### 2.2. Morphology and Texture of the Composites Obtained from Glycerol Slurries

**Figure 3:**
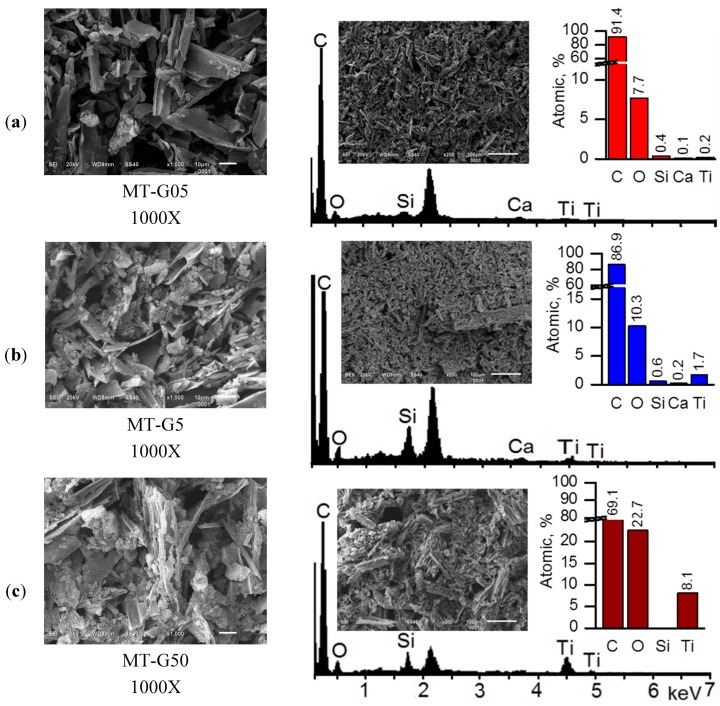
Scanning electron microscopy images of the TiO_2_/carbon composites (**left**) and EDS spectra obtained at magnification 200× for semi-quantitative analysis (**right**). MT-G05 (**a**); MT-G5 (**b**) and MT-G50 (**c**).

All of the prepared TiO_2_/carbon composites were black, and their morphologies were similar. Figure 3 shows the SEM images of the samples prepared by mixing the TiO_2_ slurry with the raw residues in glycerol suspensions (variant (b) in Figure 1): MT-G05 (a), MT-G5 (b) and MT-G50 (c). The incorporation of the TiO_2_ nanoparticles or aggregates into the striae of the *Manihot* carbon trunks was possible. The images indicated immobilization of TiO_2_ agglomerates on the surface of MT-G5 as well as the presence of a free carbonaceous surface. The surface of composite MT-G50 (Figure 3c), which contained 10-fold more TiO_2_, exhibited a crust of TiO_2_ covering the carbonaceous surface to a greater extent. For this material, we could expect near semiconductor behavior.

The surface analysis by SEM revealed that MT-G05 was comprised of TiO_2_ immobilized aggregates (<1 μm in size), and MT-G5 contained irregular aggregates ~5 μm in size. Sreethawong *et al.* also used glycerol as a mesopore-controlling agent and obtained clusters of uniform TiO_2_ nanoparticles, which is in agreement with our results [22].

**Figure 4:**
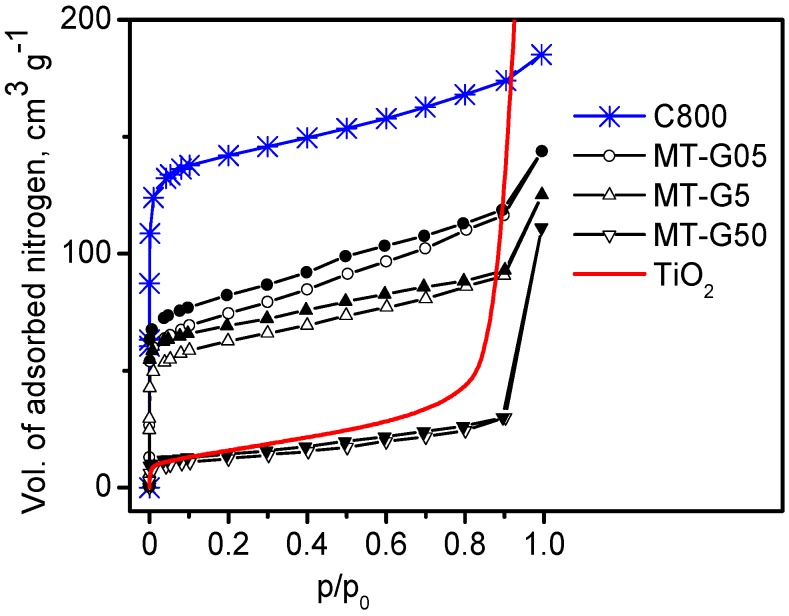
N_2_ adsorption (empty symbols)-desorption (filled symbols) isotherms at 77 K for the composites prepared by mixing slurries of the precursors in glycerol (variant (b) in Figure 1) using different residue: TiO_2_ ratios.

The loaded amount of TiO_2_ influenced the textural characteristics of the composites prepared by mixing glycerol slurries, as demonstrated by measuring the N_2_ adsorption–desorption isotherms. The specific surface area (S_BET_) and other textural parameters of the composites prepared by variant (b) (see Figure 1) are listed in Table 2. The nitrogen adsorption isotherms of samples MT-G05 and MT-G5 were significantly different from the typical isotherm for the commercial nonporous anatase TiO_2_ (Figure 4). Remarkably, the curves of both composites with low and medium TiO_2_ loading were located below the isotherm corresponding to *Manihot* carbon C800 prepared at the same temperature. The decrease (see Table 2) in the magnitude of S_BET_ (244 ± 22 m^2^·g^−1^ for low TiO_2_ loading and 203 ± 9 m^2^·g^−1^ for medium TiO_2_ loading) with respect to C800 (449 m^2^·g^−1^) was a direct result of the blockage of micropores by glycerol and TiO_2_ nanoparticles in MT-G05 and agglomerates in MT-G5.

**Table 2:** Specific surface S_BET_, micropore S_0_^t^, external surface S_e_^t^ and point of zero charge pH_pzc_ of the TiO_2_/C materials prepared by mixing the precursors in a glycerol suspension in different *Manihot*: TiO_2_ ratios according to variant (b) in Figure 1, and the corresponding isotherms are shown in Figure 4.

Composite	*Manihot*: TiO_2_ Ratio	Specific Surface, m^2^·g^−1^			V_μ_, cm^3^·g^−1^	V_tot_, cm^3^·g^−1^	*D*, nm	pH_pzc_
		BET	S_0_^t^	S_e_^t^				
MT-G05	1:0.5	244	118	126	0.060	0.204	3.6	11.4
MT-G5	1:5	203	104	99	0.053	0.178	3.8	7.7
MT-G50	1:50	40	-	40	-	0.158	26.7	6.0

The nitrogen adsorption isotherm (lowest curve in Figure 4) for sample MT-G50 with a maximum TiO_2_ load was in agreement with the isotherm of bare commercial TiO_2_ (curve without points in Figure 4). Correspondingly, the S_BET_ of composite MT-G50 (40 ± 3 m^2^·g^−1^) was similar to that of TiO_2_ (58 m^2^·g^−1^).

As expected, composites MT-G05 and MT-G5 (Figure 4) exhibited a type IIb hybrid isotherm. The hybrid isotherm was consistent with a combination of adsorption isotherms corresponding to the textural characteristics of two materials with different adsorption isotherms (*i.e.*, carbon (type I) and TiO_2_ (type II)). The isotherms of these two composites exhibited a low-pressure hysteresis (LPH), which has been observed for some microporous adsorbents. The pore size distribution of composite MT-G50 (not shown here) indicated macropores.

Samples MT-G05 and MT-G5 exhibited mesoporosity with D_p_ > 2 nm, micropores with diameters ≤2 nm and a similar micropore volume (see Table 2) due to partial blockage of the micropores. The micropore volumes of the composites with reduced TiO_2_ content were similar, and in composite MT-G50, the micropores disappeared (*i.e.*, V_μ_ = 0.160 cm^3^·g^−1^ in carbon C800, V_μ_ = 0.060 cm^3^·g^−1^ in MT-G05 and V_μ_ = 0.053 cm^3^·g^−1^ in MT-G5). However, both composites exhibited a micropore/mesopore ratio of ~50%. Velasco *et al.* demonstrated that immobilization of TiO_2_ only occurred on the outer surface of the carbon when they prepared their carbon-TiO_2_ composite by infiltration of TiO_2_ nanoparticles into commercial carbon [23]. The S_0_^t^ and S_e_^t^ values of our TiO_2_/carbon samples indicated no substantial difference in the contribution of the micropores to the magnitude of the specific surface area when the material was prepared with 0.5 and 5 mg of TiO_2_. Notably, in solid-liquid photocatalytic reactions, large reactants do not diffuse into micropores. However, the creation of micropores directly leads to an increase in the specific surface area. As shown in our work, micropores and mesopores coexist, and their ratio can be affected by the immobilized TiO_2_. The evidence for micropore (D_p_ < 2 nm) blocking by TiO_2_ nanoparticles (d_p_ < 50 nm) complements Velasco’s *et al.* demonstration of the immobilization of TiO_2_ on the outer surface of commercial carbon [23]. Furthermore, our results indicate that in addition to pore blocking, inhibition of pore formation also occurs because the formed TiO_2_ nanoparticles settled on the raw *Manihot* residues prior to carbonization, as described in this and the previous section.

Composites containing more than 20% TiO_2_ have been reported to exhibit operational problems, and nearly 100% of the phenol from the aqueous solution was removed [23]. In comparison, the TiO_2_ loadings used in our work exhibited excellent wettability and could be separated from the solution by precipitation without additional operations.

The achievement of a high surface area is a challenge in the preparation of a crystalline TiO_2_-containing photocatalyst, which is primarily due to the Degussa TiO_2_ nanoparticles being non-porous. Notably, composites MT-G05 and MT-G5 exhibited S_BET_ magnitudes much higher than that of TiO_2_ and approximately 50% microporosity. Despite the considerable number of publications on the preparation of TiO_2_ loaded carbon, the reproducibility of the textural characteristics of the resulting composites has never been reported. In the current work, four lots of each composite were prepared, and their specific surface areas, which are shown in Table 2, represent mean values with STD ≤ 9%. Based on the known error in the BET measurements, these results indicate the acceptable reproducibility of the preparation method. As previously reported [19], the photocatalytic activity is also related to the texture of the material. We succeeded in preparing two composites (*i.e.*, MT-G05 and MT-G5) that exhibit similar textural properties but different TiO_2_ loads. This fact has economic implications, and one cannot rule out that a low population of well-dispersed TiO_2_ nanoparticles can perform differently from TiO_2_ clusters (compare Figure 3a,b). The photocatalytic performance of the samples prepared by this methodology will be discussed below.

### 2.3. Surface Acidity of the TiO_2_/Carbon Samples Obtained from Glycerol Suspensions

Activated carbon is an amphoteric material with acidic and basic sites on its surface. The point of zero charge (pH_pzc_) (see Table 2) differs depending on the TiO_2_/carbon ratio. At 800 °C, we achieved sa basic carbon with a pH_pzc_ value of 11.4. Table 2 indicates that the low concentration of TiO_2_ in sample MT-G05 produced pH_pzc_ values identical to the corresponding carbon C800. Medium and high TiO_2_ loadings in composites MT-G5 and MT-G50 reflected the influence of TiO_2_ on the acidity of the composite (pH_pzc_ = 7.7 and 6.0). This result demonstrates one of the features of a composite (*i.e.*, a different acidity with respect to the composite constituents).

The surface acidity is rarely discussed in the characterization of carbon-TiO_2_ composites. Lim *et al.* remarked that high values of pH_pzc_ from carbons prepared at high temperatures (H-type activated carbon) increased electron transfer between carbon and TiO_2_ [8]. Velasco *et al.* observed a small change in the pH_pzc_ of their composite [23]. In contrast, the pH_pzc_ of the materials prepared by Matos *et al.* varied due to the carbon preparation parameters [6]. In addition, these authors demonstrated that the pH_pzc_ of the surface influenced the photocatalytic degradation of 4-chlorophenol more than the texture, reactant adsorption mechanism and intermediate product distribution [24]. Therefore, we might expect a distinct behavior in acidic/basic media and different performances of our composites in photocatalytic experiments, which will be demonstrated later.

### 2.4. Crystal Structure of TiO_2_ Immobilized on Manihot Carbon and the Structure of the Carbon

**Figure 5:**
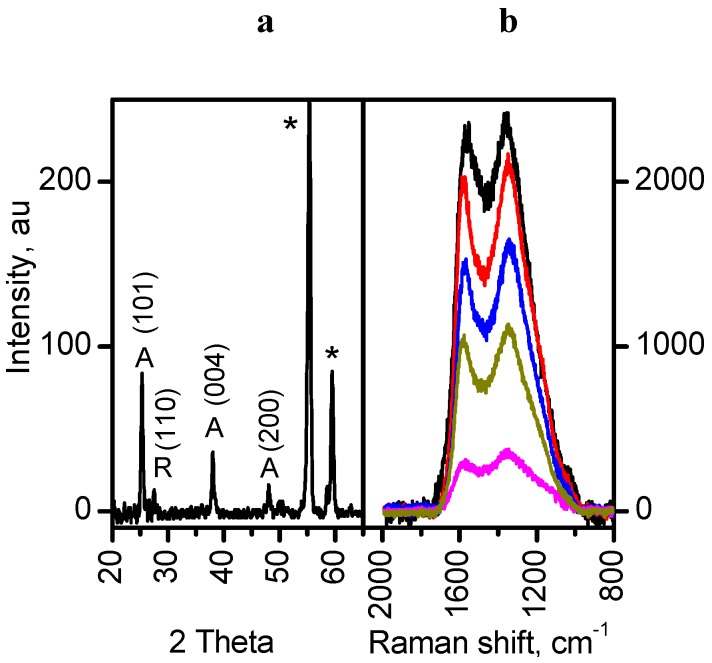
(**a**) X-ray powder diffraction patterns of sample MT-G5 showing the characteristic planes of anatase (A); (**b**) Micro-Raman spectra at five different sites of MT-G05. * = sample holder.

The phase content of P25 Degussa TiO_2_ is not consistent, and it may vary from batch to batch. Nevertheless, P25 Degussa TiO_2_ is a mixture of anatase and rutile phases with a predominance of anatase (>80%). Figure 5a shows the XRD pattern of the MT-G5 composite. Despite the low intensity of the peaks, the persistence of the anatase phase was observed (PDF 96-900-9087 from the Crystallography Open Database). The series of composites prepared in this work exhibited similar diffractograms with the main peak corresponding to the (101) plane of anatase at approximately 2θ = 25.32°.

The phase transition from anatase to rutile occurs at approximately 600 °C, but in the materials prepared at 800 °C, this phase transition did not occur. Here, the presence of glycerol may link TiO_2_ nuclei via hydrogen bonds and stabilize the anatase phase [25]. Another possible explanation is that the carbon formed in the vicinity of TiO_2_ suppresses the phase transformation at high temperature [26].

A widely accepted notion is that the anatase phase is more active than the rutile phase [27], although there are studies reporting the effectiveness of mixtures with different anatase-rutile ratios [18]. However, both phases are used in the photodegradation of pollutants. The content of the rutile phase in Degussa TiO_2_ can be observed as a very small peak at 2θ = 27.4° corresponding to the (110) plane. This peak is the most intense peak for the rutile phase, and this peak is less intense than the most intense peak for anatase in the Degussa TiO_2_. The low intensity of the peak for rutile in the diffractograms of the prepared composites may be due to the low loading of TiO_2_. In addition, the glycerol may produce linkages between the TiO_2_ nanoparticles and the raw *Manihot* residues creating a stabilizing effect against the transition of anatase to rutile during heating. If this linkage exists, this effect should occur before glycerol decomposes at 246 °C [28]. In comparison, the vascular residues of *Manihot* are completely decomposed at 400 °C [20].

For the organic residues, the carbonization temperature (800 °C) was low to ensure complete graphitization in the TiO_2_/carbon samples. Therefore, the peak at *ca*. 2θ = 25° cannot be assigned to the reflections of the (002) plane of two-dimensional graphite and was unequivocally attributed to anatase. Figure 5b shows the Raman spectra resulting from five micro-Raman measurements. These spectra contain the defect-derived D (1375 cm^−1^) and the graphite structure-derived G (1550 cm^−1^) bands, which are characteristic of carbonaceous materials that are specific for each material. The prepared composites exhibited a large D-band peak (I_D_/I_G_ ~ 2.7), indicating an amorphous carbon content of approximately 75%. Because only slightly lower values were obtained in the absence of glycerol (I_D_/I_G_ ~ 2.4 and the amorphism I_D_/(I_D_ + I_G_) ~ 70%), this additive did not substantially contribute to the amorphism of the carbonaceous component in the TiO_2_/carbon materials.

### 2.5. Adsorption of IC

**Figure 6:**
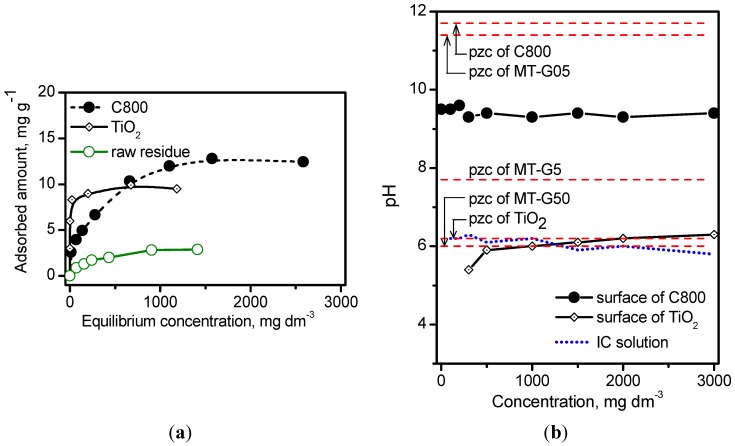
(**a**) Adsorption isotherms of IC on raw *Manihot* residue, TiO_2_ and carbon C800 at 25 °C. Adsorbent dose: 33.3 mg·cm^−3^; (**b**) Variation in the pH of the IC solution as well as of the surface of TiO_2_ and the carbon C800 after adsorption of different concentrations of IC. The levels of pH_pzc_ of the materials are denoted with dashed lines.

Figure 6 shows the isotherms describing the adsorption of IC onto the raw *Manihot* residues, carbon sample C800 and commercial TiO_2_ without any adjustment. The Degussa TiO_2_ used in this work exhibited a saturation capacity of 0.20 molecules·nm^−2^ compared to 0.13 molecules·nm^−2^ reported by Vautier *et al.* [29] for an adsorbent dose of 2.5 mg·cm^−3^. Alahiane *et al.* [30] reported a saturation capacity of 0.21 molecules·nm^−2^ for an adsorbent dose of 1.0 mg·dm^−3^. Carbon C800 exhibited a slightly higher saturation capacity of 0.27 molecules·nm^−2^. The corresponding Langmuir constant (k_L_) was calculated by the non-linear form of the Langmuir equation, and these constants were 189 L·mg^−1^ on the raw residue, 0.4 L·mg^−1^ on TiO_2_ and 256 L·mg^−1^ on carbon C800.

Once the relative adsorption affinity of IC on the semiconductor TiO_2_ and carbon was determined, we explored the acidity/basicity of the drained surface immediately after adsorption. The dotted line in Figure 6 indicates that there is no variation in the pH, within the experimental error, with the IC concentration. The pH on the surface of the wet composites was measured after the adsorption process. The pH of the TiO_2_ surface remained near the values of the IC solution and near the pH_pzc_ of TiO_2_ at all of the concentrations. In contrast, the pH of the carbon C800 surface decreased from a pH_pzc_ value of 11.7 to 9.5 because the dye acted as a neutralizing agent. As previously discussed, the results in Figure 6b indicate that the pH_pzc_ of MT-G05 resembles that of carbon C800, the pH_pzc_ of MT-G50 resembles that of Degussa TiO_2_, and the pH_pzc_ of MT-G5 is midway between carbon and TiO_2_. The results in Figure 6b suggest that the adsorption of IC (pK_a_ 12.2) in basic medium (pH > 8) should only occur on composite MT-G05 because pH ˂ pH_pzc._ In an acidic solution (pH ˂ 6), the adsorption occurs on all the composites because pH ˂ pH_pzc_.

### 2.6. Photocatalytic Decomposition of IC

TiO_2_/carbon materials can exhibit enhanced photoactivity because the synergistic effect that causes a rapid photoinduced charge separation and a diminution of the recombination of the electron-hole pairs in TiO_2_. To better estimate the photoactivity of a series of samples, photoelectrochemical analysis offers an excellent opportunity to assess differences in the transfer of photogenerated electron-hole pairs from TiO_2_. Zhang *et al.* demonstrated that the higher and more stable photocurrent of the sample, the higher photoactivity and they showed the importance of the carriers lifetime in graphene-TiO_2_ [31].

The photocatalytic performance test of carbon-TiO_2_ materials produced using biomass as carbon source has involved methylene blue [14], Rhodamine B [15], basic red 18 and basic red 46 [17]. The decay of the TOC [14] and COD [15] values of the dye solutions was faster on the carbon-TiO_2_ materials than on TiO_2_. However, this result could be related with an adsorption effect. In contrast, Mahmoodi *et al.* demonstrated that two azo dyes degraded faster using TiO_2_ immobilized on activated carbon in comparison to TiO_2_ [17]. Total mineralization occurred after 80–100 min irradiation at 200–280 nm. One of the purposes of the current paper was to evaluate the performance of our TiO_2_/carbon materials for the photocatalytic decomposition of IC. Research conducted for this purpose has been reported by Subramani *et al.* [32] by mixing both commercial TiO_2_ and carbon (S_BET_ 1025 m^2^·g^−1^) in HNO_3_ followed by heating in an autoclave at 150 °C. Recently, some novel visible-light photocatalytic materials have also been tested for photodegradation of IC [33]. However, in contrast to those doped semiconductors, we investigated the simple preparation of a TiO_2_/carbon composite to observe the adsorption and photocatalysis phenomena during the removal of IC.

**Figure 7:**
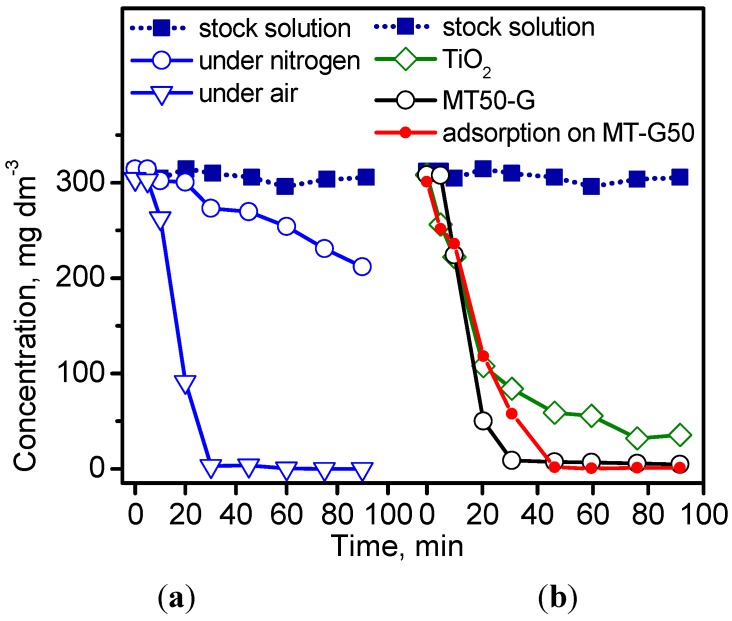
Kinetic curves of (**a**) photolysis at 50 °C in a nitrogen and air atmosphere and (**b**) photocatalysis of the dye IC at 50 °C in an air atmosphere. Adsorption experiment of IC on MT-G50 in the dark.

Figure 7 shows the concentration decay of the irradiated IC solutions at 254 nm after photolysis (Figure 7a) and photocatalysis (Figure 7b). As expected [34], IC decomposed by photolysis and more readily in air atmosphere due to the presence of oxygen (Figure 7a). Surprisingly, the prepared TiO_2_/C MT-G50 sample exhibited superior elimination efficiency compared to TiO_2_ (Figure 7b) due to the adsorption capacity of the uncovered segments of the carbonaceous support in the composite.

As shown in Figure 7b, carbon C800 completely adsorbs the dye after 40 min of contact. In addition, the carbon C800 reached saturation with 14 mg·g^−1^ of IC, and the specific surface decreased from 434–346 m^2^·g^−1^ for the saturated adsorbent [35]. However, the adsorption of IC on C800 was reversible because after contact with water, the original magnitude of S_BET_ was reestablished (*i.e.*, 437 m^2^·g^−1^).

**Figure 8:**
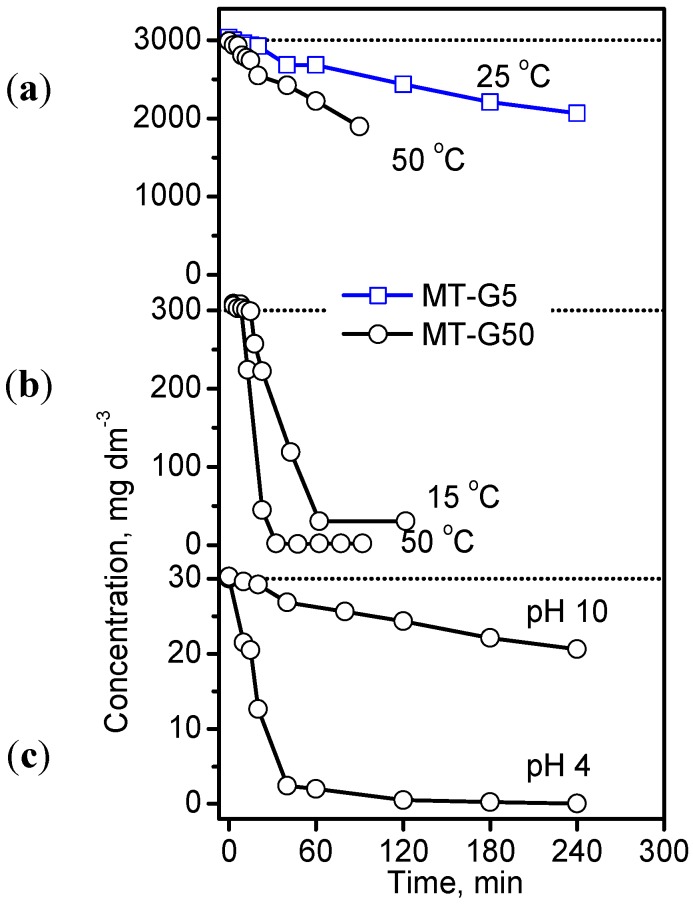
Photocatalytic decomposition of the IC dye with three concentration levels at natural pH and different temperatures (**a**,**b**) as well as at 25 °C in basic and acidic solutions (**c**) using two composites in an air atmosphere.

Since the study of Vautier *et al.* was reported, the photocatalytic degradation of IC as model indigoid dye has been studied extensively via UV irradiation [29]. The most widely used photocatalysts are TiO_2_, doped TiO_2_, and TiO_2_ containing composites. Barka *et al.* [36] demonstrated that the apparent degradation rate constant of IC was affected by the temperature, concentration, pH, and previous adsorption in the dark. After demonstration of the adsorption affinity between IC and the constituents of our TiO_2_/carbon composites, the photocatalytic degradation of the dye under different conditions was explored. Figure 8 shows the decrease in the concentration of IC as a function of time at different temperatures and pH values for IC initial concentrations varying by two orders of magnitude using the photocatalysis conditions provided in the experimental section. The study of other factors influencing the photodegradation process, such as the volume of the IC solution and the mass of the photocatalyst, were excluded because they involve the design of the photoreactor. By comparing the results in Figure 8a with those in Figure 8b,c, we observed that an extreme initial concentration of 3000 mg·dm^−3^ exhibited the slowest degradation rate independent of the composite and temperature. Despite the large differences in the initial concentrations used by us and other authors [31,36,37], the effect was similar. The kinetic curves in Figure 8a obeyed a second order rate equation, and the rate constants were 0.5 × 10^−2^ for MT-G5 at 25 °C and 1.5 × 10^−2^ g·mg^−1^·h^−1^ for MT-G50 at 50 °C. Using a concentration of 300 mg·dm^−3^ (Figure 8b), 90% degradation was achieved after 60 min of irradiation, and this degradation occurred more readily at 50 °C. The rate constant (*k_2_*) at 25 °C was 8.4 × 10^−2^ g·mg^−1^·h^−1^ using MT-G50 and 0.5 × 10^−2^ g·mg^−1^·h^−1^ using MT-G5. Alahiane *et al.* observed a first order degradation rate of 2 × 10^−2^ min^−1^ for 20 mg·dm^−3^ IC using Degussa TiO_2_ [30], and Barka *et al.* obtained a first rate constant of 3 × 10^−2^ min^−1^ using fibers coated with PC500 TiO_2_ [36]. However, Barka *et al.* reported an unsatisfactory first order kinetic behavior at higher initial concentrations and suggested a competing adsorption/degradation mechanism.

The results in Figure 8c indicate that IC degradation occurred slowly at pH 10 using composite MT-G50 with a pH_pzc_ of 6.0, (see Figure 6b) where the surface is negatively charged resulting in repulsion of the dye molecule. In contrast, a pH of 4.0 favored the attraction between the positively charged surface and the dye, as discussed in the previous section. These results agree with those obtained for the adsorption of IC on TiO_2_ PC500 [36].

In addition, we decided to optimize the photodegradation of IC using the prepared TiO_2_/carbon materials. Abaamrane *et al.* reported on the optimal decolorization of IC using TiO_2_/UV and the response surface methodology [37]. In the current work, we used the Taguchi method [38] for the evaluation of the color removal produced by IC through a small number of trials. This technique included transformation to a signal to noise ratio (S/N), which is a measure of the variations observed. An L_4_ orthogonal array was applied, and the three factors selected included the specific surface (40 m^2^·g^−1^ in MT-G50 and 203 m^2^·g^−1^ in MT-G5), initial concentration (30 and 300 mg·dm^−3^) and temperature (25 and 50 °C). The pH of the solution was maintained at 4 for all of the experiments because (i) composite MT-G05 was excluded due to a pH_pzc_ value that was very close to that of its precursor carbon C800. The removal involves both adsorption and photocatalysis occurring on prepared composites MT-G5 and MT-G50 with different TiO_2_ loadings, the representative factor describing their whole removal capacity was the specific surface, which was determined to be 203 m^2^·g^−1^ for MT-G5 and 40 m^2^·g^−1^ for MT-G50; (ii) As demonstrated in Figure 8c, the performance of MT-G50 at a pH of 4 was much better than that at a pH of 10. Therefore, according to Figure 6b, the selected materials were used in acidic solutions of the IC dye, and the pH was not used as a factor in the optimization study. The array of experimental factors with two levels and the results are reported in Table 3. The extent of decolorization varied from 10%–99% in the different combinations.

**Table 3:** Experimental L_4_ Taguchi orthogonal array with the color percentage removal as an output variable. Factors and levels: A: S_BET_ (40, 203 m^2·^g^−1^); B: concentration (30, 300 mg·dm^−3^); C: temperature (25, 50 °C).

Factor Levels			Color Removal, %		S/N	Removed TOC, %
A	B	C	Exper. 1	Exper. 2		
1	1	1	10	16	18	61
1	2	2	99	98	40	55
2	1	2	94	94	39	54
2	2	1	40	37	31	48

The array indicated that the temperature was the most influential factor (S/N = 40), and an experiment to confirm these results was carried using the optimal level of each factor (combination A2,B2,C2), and this experiment produced 95% removal (T = 50 °C, initial concentration = 300 mg·dm^−3^ and specific surface = 203 m^2^·g^−1^ of the composite MT-G5). The specific surface influenced the removal of the dye rather than the amount of TiO_2_ loaded on the materials. Regarding the decolorization, the elimination of the total organic carbon was slightly lower for a high initial concentration. For example (see last column in Table 3), a 55% TOC removal was observed using an initial concentration of 300 mg·dm^−3^ compared to a 61% TOC removal using 30 mg·dm^−3^. The inconsistency between the high removal of color (e.g., 94% in the combination A2,B1,C2) and the 54% removal of TOC is due to the formation of uncolored products, and these products identification will be discussed in the next section.

### 2.7. Identification of the Photodegradation Products of IC

**Figure 9:**
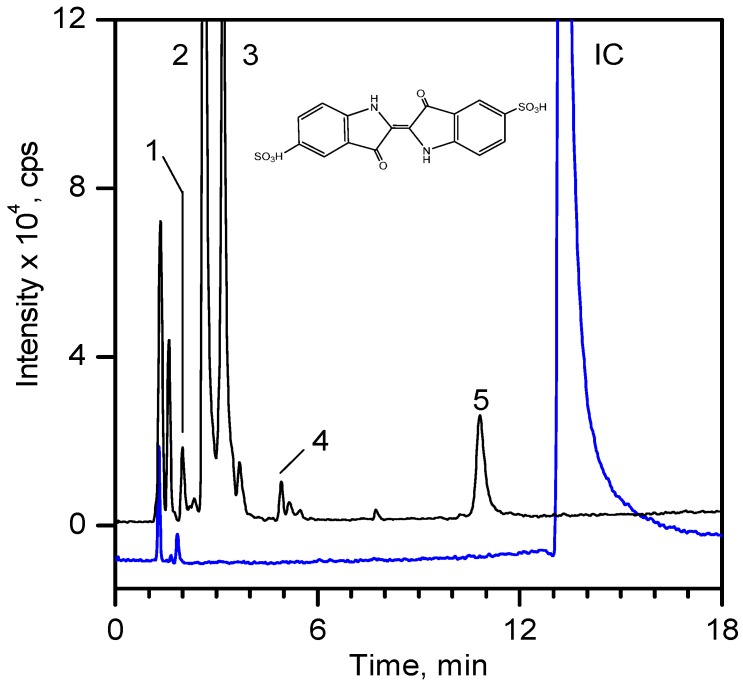
LC-MS chromatogram of the stock solution of IC (300 mg·dm^−3^) and a selected reaction mixture after 60 min of photocatalytic reaction using composite MT-G5 (200 m^2^·g^−1^) at a pH of 10 and 15 °C. The peak numbering is shown in Table 4.

For product identification, we monitored selected aliquots of the IC solutions subjected to the photocatalytic experiments. Figure 9 shows the LC-MS chromatograms of the stock solution of IC and one reaction mixture. Table 4 lists the retention times, exact masses of the molecular ions (*m*/*z*), error values (Δ*m*/*z*), and the proposed structure of the products detected in Figure 9.

**Table 4:** Retention time and possible structures identified by LC-ESI-Qq-TOF-MS as products of IC during the photocatalytic reaction at 254 nm. The references reporting the same compound are given in brackets.

**Proposed structure**	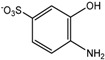	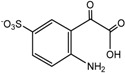	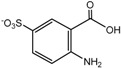
Name	4-amino-3-hydroxybenzenesulfonic acid	2-(2-amino-5-sulfophenyl)-2-oxoacetic acid	2-amino-5-sulfobenzoic acid
**Peak nr.**	**1**	**2** [39,40,41]	**3** [39,40]
*t*_R_ (min)	2.00	2.63	3.20
Formula	C_6_H_7_NO_4_S	C_8_H_7_NO_6_S	C_7_H_7_NO_5_S
*m*/*z*_exper_	188.0032	243.9924	215.9981
*m*/*z*_calc_	188.0023	243.9921	215.9972
Δ*m*	4.79	1.23	4.17
**Proposed structure**	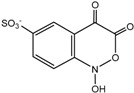	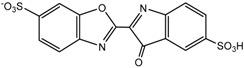	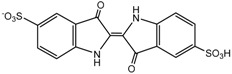
Name	1-hydroxy-3,4-dioxo-3,4-dihydro-1H-benzo[c][1,2]oxazine-6-sulfonic acid	2-(3-oxo-6-sulfo-3H-indol-2-yl)benzo[d]oxazole-6-sulfonic acid	(E)-3,3’-dioxo-[2,2’-biindo-linylidene]-5,5’-disulfonic acid
**Peak nr.**	**4**	**5**	**IC**
*t*_R_ (min)	4.93	10.8	13.23
Formula	C_8_H_5_NO_7_S	C_15_H_8_N_2_ O_8_S_2_	C_16_H_10_N_2_ O_8_S_2_
*m*/*z*_exper_	257.9717	406.9653	420.9817
*m*/*z*_calc_	257.9714	407.9649	420.9806
Δ*m*	1.16	0.98	2.61

At least 6 new peaks eluted prior to the appearance of the dye peak in the aliquot chromatogram, and all of cases carried out under the Taguchi conditions exhibited the same products in different proportions. The mass error accuracy of the proposed structures was less than 5 ppm. In addition, the molecular ions with *m*/*z* 244 (peak 2) and 216 (peak 3) are the most abundant products and coincide with the compounds formed by photocatalysis on SnO_2_ [39] and photoelectrocatalysis on Ti/TiO_2_/WO_3_ electrodes [40]. Interestingly, under visible-light irradiation, the ZnBiSbO_4_ photocatalyst produced 2-amino benzoic acid, which is the non-sulfonated form of compound 2 in Table 4 [42]. Electrochemical [43] and biological degradation processes [41] have also been reported to produce the aromatic compound isatin-5-sulfonic acid (mass 226) as an important degradation product of IC. We did not detect this product from the oxidative breaking of the indigoid C=C bond under any experimental condition. In contrast, we identified 2-(2-amino-5-sulfophenyl)-2-oxoacetic acid (peak 2, *m*/*z* 243.9924), which easily forms from isatin-5-sulfonic acid by addition of H_2_O [44].

In addition to the sulfonated amines (peaks 1–3 in Table 4) bearing only one phenyl ring, the structures proposed for two of the other products (*i.e.*, peaks 4 and 5 in Figure 9) are given in Table 4. The oxazine compound (peak 4), which has not been previously reported, was confirmed by MS/MS yielding the following product ions: 194 [258-SO2]^−^, 178 [258-SO3]^−^, 160 [258-H2SO4]^−^, 150 [258-C6H4O2]^−^, 133 [258-H3O5S]^−^, 123 [258-CHO5S]^−^, and 80 [258-C8H4O4N]^−^.

## 3. Experimental Section

### 3.1. Materials

Aeroxide P25 titanium oxide (batch number 1036070711; Degussa, Parsippany, NJ, USA) and glycerol (GR, 87%; Merck, Darmstadt, Germany) were used as the starting reagents. The average particle size certified by the producer was 25 nm. The residues obtained from the vascular system of *Manihot* stems have previously been thoroughly characterized by our group [20] and were used with a constant moisture value of 50% ± 4%. Indigo carmine (C.I. 73015) was purchased from Sigma-Aldrich (St. Louis, MO, USA).

### 3.2. Samples Preparation

The yield of vascular system residues after removal of the rind and pith was 55% weight from the *Manihot* stems. The residues were passed through a sieve to obtain a particle size of 0.45 mm. The particles density was 0.1 g·cm^−3^. Two variants of the preparation method were used: (a) Raw *Manihot* residues were mixed manually with dry commercial TiO_2_ (1:1 weight ratio) for subsequent wetting with water or glycerol (10 cm^3^) to produce samples MT, MT-W and MT-G; and (b) a glycerol slurry of raw *Manihot* residues was mixed with an aqueous suspension of commercial TiO_2_ for subsequent carbonization to fabricate samples MT-G05, MT-G5 and MT-G50. The composites of this series were prepared using the raw *Manihot* residues, water, glycerol and TiO_2_ nanoparticles in different ratios. Slurries of different quantities of TiO_2_ in 10 cm^3^ of water were added to a suspension of 10 g of the raw *Manihot* residues in 10 cm^3^ of glycerol for the different composites and stirred. The mass of the residues was obtained by considering the carbon yield, which was 25% by weight from the raw *Manihot* residues. The resulting homogeneous suspensions were maintained in a combustion boat at room temperature for 24 h. A horizontal tubular furnace (Carbolite, Ubstadt-Weiher, Germany) with a quartz tube was used to heat the composites from room temperature to 800 °C. The temperature regime was designed according to the thermal behavior of the residues, as reported in our previous publication [20]. This regime consisted of two heating ramps involving heating from room temperature to 50 °C at 4 °C·min^−1^, holding at 50 °C for 1 h and heating from 50 to 800 °C at 4 °C·min^−1^. The materials were maintained at the maximum temperature in the carbonization atmosphere for 4 h. Then, the oven was cooled to room temperature. The sample labels correspond to the carbon: TiO_2_ weight ratios and the use of water (W) or glycerol (G), as shown in the scheme in Figure 1.

### 3.3. Characterization of the Materials

The morphology of the samples was determined using a JEOL JSM 6610 LV scanning electron microscope (SEM) (Peabody, MA, USA). The EDS analysis was performed with a microprobe Quest Noran sonde coupled to the microscope. The micrographs were recorded at 15 kV, and the samples were prepared by gold sputtering.

The BET surface area was measured by nitrogen adsorption at 77 K using an Autosorb-1 instrument from Quantachrome (Boyton Beach, FL, USA) after outgassing at 573 K for 24 h. The specific surface area was calculated using the Brunauer–Emmett–Teller (BET) equation. The extent of microporosity and mesoporosity was calculated using the V-t method and the micropore volume obtained from the nitrogen adsorption isotherms. All of the nitrogen adsorption data were evaluated according to standard gas adsorption research procedures. The reproducibility of the preparation of several lots was tested by measuring 11 adsorbed volumes in the range 5 × 10^−2^ ≤ p/p_0_ ≤ 3 × 10^−1^ and applying the BET equation.

The pH of the point of zero charge (pH_pzc_) of the raw residue and the carbon was measured by potentiometric titration. For this purpose, 10 mg of the residue particles was stirred for 24 h in 3 cm^3^ of solutions with different pH_initial_. Then, the final pH was measured with a Titrando Metrohm and plotted as a function of pH_initial_, and the pH_pzc_ was determined as the point where pH_final_ = pH_initial_. For the TiO_2_-carbon composites, the pH_pzc_ values were determined according to the method of potentiometric mass titration [45].

X-ray diffraction (XRD) measurements were carried out using a D8 Advance diffractometer from Bruker AXS (Karlsruhe, Germany) with CuKα_1_ radiation (α = 1.5406 Å). The data were collected over a 2θ range of 10°–70° with a step size of 0.026° and a measurement time of 43.2 s/step. The micro-Raman spectra were measured using a high-resolution dispersive Jobin Yvon-Horiba (Villeneuve d’Ascq, France) LabRAM HR model spectrophotometer equipped with a 632.8 nm (17 mW) He-Ne laser and a Nikon BX41 microscope with a 100× objective and a resolution of 1 cm^−1^.

### 3.4. Adsorption of IC from Aqueous Solution

Adsorption tests were performed at 25 °C in thermostated batch experiments. The stem residues (d_p_ 0.125 mm), carbon, and TiO_2_ were dried for 24 h at 75 °C. Then, 50 mg of each material was weighed into polycarbonate cylindrical cells with a lid and placed in contact with 1.5 cm^3^ of an IC solution with concentrations in the range of 100–1500 for the raw residues and 100–3000 mg·dm^−3^ for TiO_2_ and carbon C800. The pH was not adjusted and was measured to be 6.0 in deionized water. The solution that resulted from the adsorption equilibrium obtained after 24 h was separated from the exhausted adsorbent and centrifuged (12,000 rpm) followed by analysis with a UV-Vis Beckman DU 7500 spectrophotometer. A ten point calibration curve was constructed at 610 nm in the concentration range of 100–1500 mg·dm^−3^ with a determination coefficient (*R*^2^) of 0.999. In addition, the pH of the solutions and drained materials was measured using a Tritando Metrohm 809 (Riverview, FL, USA) and the LL Biotrode and LL flat membrane electrodes, respectively.

### 3.5. Photocatalytic Experiments

For the irradiation experiments, a 15 cm^3^ temperature-controlled quartz reactor from Ace Glass Inc. (Vineland, NJ, USA) was used. A 2⅛-inch Pen-Ray light source (254 nm, 5.5 Watt) from Ultra-Violet Products (Upland, CA, USA) was immersed into the reactor in a quartz jacket. The reactor was equipped with a water jacket to avoid IR irradiation of the lamp and control the temperature (25 °C) of the solution. The entire assembly was enclosed in a dark chamber, and the lamp was switched on after 30 min to establish adsorption equilibrium. In each experiment, 10 mg of the composite was added to 5 cm^3^ of a solution containing 3000, 300 or 30 mg·dm^−3^ of IC. Because the particles of the prepared material settled readily, no centrifugation step was required to collect aliquots at regular time intervals during irradiation for spectroscopic and chromatographic analysis, which is in contrast with studies using nude anatase nanoparticles. The pH of the IC solutions was adjusted in selected experiments with 0.5 N NH_4_OH to achieve a pH of 10 and with 0.025 N HCl to achieve a pH of 4.0.

### 3.6. Analysis of Photocatalysis Products

Aliquots of all of the IC solutions subjected to photocatalytic testing with the TiO_2_-carbon composites were analyzed using a Hach DR 5000 UV-Vis spectrophotometer over a wavelength range of 200–800 nm. Decolorization was evaluated at 610 nm. The collected aliquots were also analyzed by liquid chromatography using an LC-MS instrument (Series 1260 chromatograph coupled with an ESI-(Qq)-TOF-MS 6520 detector from Agilent Technologies, Santa Clara, CA, USA). The separation was carried out at 25 °C using a Nucleodur EC C18 Isis (5 µm, 250 × 4.6 mm) column from Macherey–Nagel (Düren, Germany). Gradient elution at 1 cm^3^·min^−1^ with a methanol-water (10%–50%) mobile phase between 0 and 20 min and 90% methanol up to 25 min was performed. The water contained 40 mM acetic acid-triethylamine reagent from Fluka (Buchs, Switzerland) as an ion pair agent. To methanol (Burdick-Jackson, Muskegon, MI, USA), 0.1% HCOOH from Aldrich (St. Louis, CA, USA) was added. The injection volume was 100 µL.

ESI-TOF-MS was performed in the negative ionization mode (ESI(-)) with nitrogen as the drying gas at 11 dm^3^·min^−1^ using a TOF fragmentor voltage of 175 V in the negative ionization mode, a capillary voltage of 3500 V over a *m*/*z* range of 50–700, gas temperature of 350 °C and a nebulizer pressure of 60 psi. For the MS/MS analysis, ESI-Qq-TOF-MS was used in the negative ionization mode using a CE of 30 V over an *m*/*z* range of 50–300 and an isolation width of 1.3 *m*/*z*.

## 4. Conclusions

The preparation method of the TiO_2_/carbon materials containing the photocatalytically active anatase phase was simple and inexpensive. The glycerol additive allowed for the conservation of the anatase phase despite treatment at 800 °C. The composites were fibrous trunks with improved density. The use of dry precursors produced mesoporous materials, but the reproducibility of their properties was low. The glycerol-aided fabrication of the TiO_2_/C composite led to optimal immobilization of TiO_2_. A low *Manihot*: TiO_2_ ratio (1:0.5) yielded a basic material with S_BET_ 244 m^2^·g^−1^, ~50% microporosity and an average pore diameter of <4 nm. The moderate TiO_2_ loading in composite MT-G5 (203 m^2^·g^−1^) also generated a basic material with the same microporosity percentage and average pore diameter. The TiO_2_ loading on the carbon directly affected the acidity (pH_pzc_) of the TiO_2_/carbon composites. The photodegradation of IC was complete, and most of the products were produced due to cleavage of the exocyclic C=C. Some products were persistent because they remained at the end of the photocatalytic process applied using the prepared composite. The identified degradation products of IC are similar to those reported by other electrolysis and advanced oxidation processes with exception of a new oxazine compound. Due to its density and topography, the prepared composites allowed for the identification of the products during the photoreaction avoiding a long centrifugation step. The study of the toxicity of the reaction mixtures is a logical extension of the current work.
